# PERcutaneous transhepatic CHOLangioscopy using a new single‐operator short cholangioscope (PERCHOL): European feasibility study

**DOI:** 10.1111/den.14697

**Published:** 2023-10-31

**Authors:** Enrique Pérez‐Cuadrado‐Robles, Simon Phillpotts, Michiel Bronswijk, Claudio Cim Conrad, Cecilia Binda, Laurent Monino, Kirill Basiliya, Marcus Hollenbach, Apostolis Papaefthymiou, Hadrien Alric, Lucille Quénéhervé, Alessandro Di Gaeta, Mathieu Pioche, Aria Khani, Diane Lorenzo, Tom G. Moreels, Gabriel Rahmi, Tom Boeken, Carlo Fabbri, Frédéric Prat, Wim Laleman, Christophe Cellier, Schalk Van der Merwe, George Webster, Mark Ellrichmann

**Affiliations:** ^1^ Department of Gastroenterology, Georges‐Pompidou European Hospital APHP.Centre Paris France; ^2^ University of Paris‐Cité Paris France; ^3^ Department of Interventional Radiology Georges‐Pompidou European Hospital Paris France; ^4^ Gastroenterology Department University and Regional Hospital Centre Brest Brest France; ^5^ Gastroenterology and Endoscopy Unit, Edouard Herriot Hospital Hospices Civils de Lyon Lyon France; ^6^ Digestive Endoscopy Department, Beaujon Hospital APHP.Nord Clichy France; ^7^ Department of Gastroenterology University College London Hospitals London UK; ^8^ Department of Gastroenterology Royal Free Hospital London UK; ^9^ Department of Gastroenterology and Hepatology, University Hospital Gasthuisberg University of Leuven Leuven Belgium; ^10^ Department of Gastroenterology and Hepatology Imelda Hospital Bonheiden Belgium; ^11^ Department of Gastroenterology Cliniques Universitaires St. Luc, Université catholique de Louvain Brussels Belgium; ^12^ Department of Interdisciplinary Endoscopy, Medical Department 1 University Hospital Schleswig‐Holstein, Campus Kiel Kiel Germany; ^13^ Division of Gastroenterology, Medical Department II University of Leipzig Medical Center Leipzig Germany; ^14^ Medizinische Klinik B, Universitätsklinikum Münster Münster University Münster Germany; ^15^ Gastroenterology and Digestive Endoscopy Unit Forlì‐Cesena Hospitals, AUSL Romagna Ravenna Italy

**Keywords:** biliary stone, biliary stricture, cholangioscopy, PTBD

## Abstract

**Objectives:**

A new short device for percutaneous endoscopic cholangioscopy was recently developed. However, feasibility and safety has not yet been evaluated. The aim of this study was to assess clinical success, technical success, and adverse events (AEs).

**Methods:**

This observational multicenter retrospective study included all patients who underwent percutaneous cholangioscopy using a short cholangioscope between 2020 and 2022. The clinical success, defined as the complete duct clearance or obtaining at least one cholangioscopy‐guided biopsy, was assessed. The histopathological accuracy, technical success, and the AE rate were also evaluated.

**Results:**

Fifty‐one patients (60 ± 15 years, 45.1% male) were included. The majority of patients had altered anatomy (*n* = 40, 78.4%), and biliary stones (*n* = 34, 66.7%) was the commonest indication. The technique was predominantly wire‐guided (*n* = 44, 86.3%) through a percutaneous sheath (*n* = 36, 70.6%) following a median interval of 8.5 days from percutaneous drainage. Cholangioscopy‐guided electrohydraulic lithotripsy was performed in 29 cases (56.9%), combined with a retrieval basket in eight cases (27.6%). The clinical success was 96.6%, requiring a median of one session (range 1–3). Seventeen patients (33.3%) underwent cholangioscopy‐guided biopsies. There were four (7.8%) cholangioscopy‐related AEs (cholangitis and peritonitis). Overall, the technical success and AE rates were 100% and 19.6%, respectively, in a median follow‐up of 7 months.

**Conclusion:**

Percutaneous endoscopic cholangioscopy with a new short device is effective and safe, requiring a low number of sessions to achieve duct clearance or accurate histopathological diagnosis.

## INTRODUCTION

Percutaneous transhepatic biliary drainage (PTBD) can be indicated in patients with previous failure of endoscopic retrograde cholangiopancreatography (ERCP) or those involving a surgically altered anatomy. In selected cases, such as patients with biliary stones or indeterminate strictures,^1^ percutaneous cholangioscopy can also be performed through the percutaneous fistula,^2^ guiding further therapies (e.g. cholangioscopy‐guided lithotripsy or biopsies) or a 13F sheath.^3^ The alternatives in such cases with altered anatomy are enteroscopy‐assisted ERCP (EA‐ERCP), endoscopic ultrasound (EUS)‐guided therapies, or surgery.

Although EUS‐guided biliary drainage (EUS‐BD) is gaining ground and should be preferred over PTBD after failed ERCP in malignant distal biliary obstruction or inoperable patients according to recent European guidelines,^4^ percutaneous cholangioscopy remains a valuable option. Especially in benign pathology, in those cases with a previous PTBD, or when EUS‐BD experience is lacking, PTBD should be considered. Moreover, a recent meta‐analysis indicates a comparable efficacy between PTBD and EUS‐BD but fewer complications in the EUS group. However, the heterogeneity was very high,^5^ even in the included randomized clinical trials. Regarding patients with an altered anatomy, a multidisciplinary decision‐making strategy can help to select the best approach based on the patient's anatomy, indication, and local expertise. There are more and more data supporting EUS‐directed transgastric ERCP in patients with a bypass surgery^6^, ^7^ and EUS‐guided gastroenterostomy in patients with afferent limb syndrome.^8^ Furthermore, EA‐ERCP can also be proposed in patients with favorable anatomy,^9^ as this technique seems less invasive than EUS‐BD and has been associated with fewer adverse events (AEs). Finally, EUS‐guided hepaticogastrostomy combined with antegrade direct peroral cholangioscopy^10^ also represents an alternative. However, all these techniques require a dedicated experience and are not broadly available.

Percutaneous cholangioscopy can be indicated as a first line in patients with altered anatomy and previous liver surgery, right liver strictures or lesion, EUS‐BD failure, or presenting with a long afferent limb leading to difficult EA‐ERCP, depending on local practices. A retrospective series^11^ including 13 patients with surgically altered anatomy who underwent 19 procedures reported a technical success of 100%, with a bile duct clearance after a median of two procedures. Similarly, combined radiological‐endoscopic management of difficult bile duct stones has been reported^12^ using percutaneous transhepatic cholangioscopy. An observational study^13^ reported a technical and clinical success of 96%, with a 100% histopathology accuracy in indeterminate biliary strictures and a 10.7% AE rate.

Most percutaneous procedures in the literature have been performed using ultraslim endoscopes^14^, ^15^ or Spyglass systems (Boston Scientific, Watertown, MA, USA), actually intended for transoral approaches.^11^, ^13^, ^16^, ^17^ However, data assessing dedicated percutaneous cholangioscopes are lacking, and there is no consensus on how to perform the technique. Recently, several case reports using a new short device (Spyglass Discover, Boston Scientific) have been published with favorable outcomes.^18^, ^19^ This new scope has specific characteristics that could increase the clinical success while decreasing the AEs and procedure times. The aim of this multicenter pilot study is to assess the feasibility, safety, and clinical outcomes of this new technology, with a focus on the technical steps of the procedure.

## METHODS

### Patients

This was an observational and multicenter retrospective study. All patients who underwent percutaneous cholangioscopy using the Spyglass Discover system from January 2020 to September 2022 were included. Those patients who underwent percutaneous cholangioscopy using other devices (e.g. Spyglass DS2, slim endoscopes), surgical cholangioscopy, or ERCP‐assisted cholangioscopy were not considered. The protocol was submitted to the Local Ethical Committee (CERUPHO) according to the Declaration of Helsinki.

Age, sex, and demographic variables were noted. The biliopancreatic anatomy of the patients, the indications of the procedure, and the presence of acute cholangitis were collected. The personal history of liver resection was also noted.

### PTBD procedure

The PTBD procedure was performed according to local practices using both internal–external and external biliary drains as previously described.^20^ The access to the percutaneous drain, the diameter of the catheter, the time from the PTBD to the endoscopic procedure, and the drain exchange prior to cholangioscopy were collected. Patients with technical failure of PTBD drainage were not considered.

### Percutaneous endoscopic procedure

**Figure 1:**
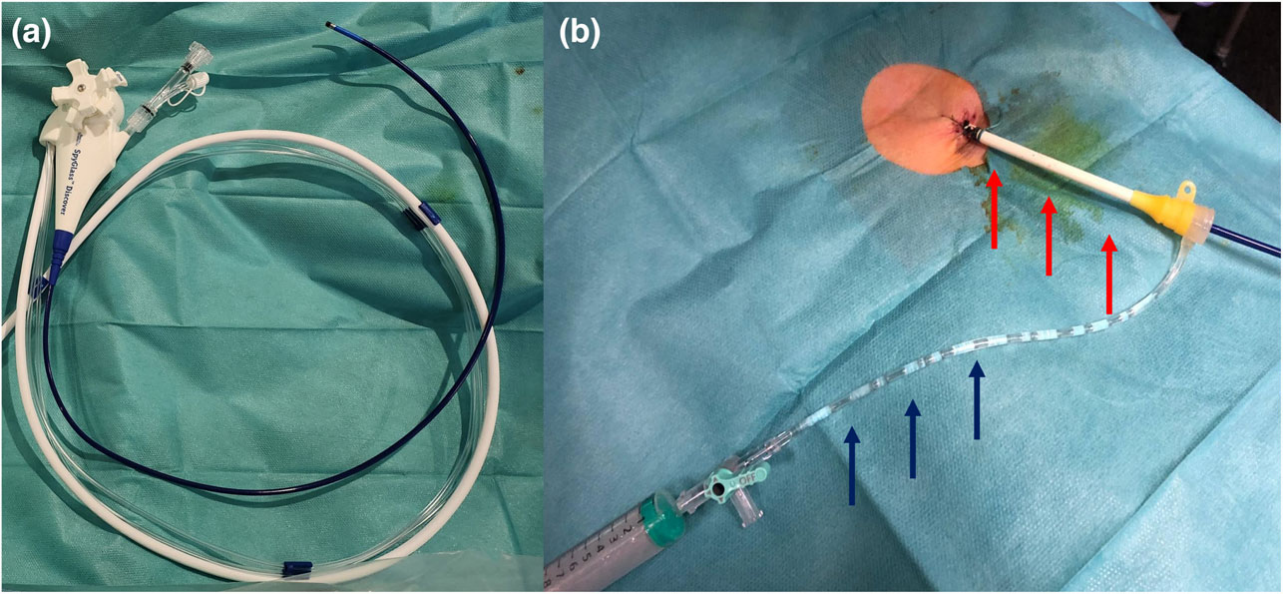
Percutaneous endoscopic cholangioscopy using Spyglass Discover (Boston Scientific, Watertown, MA, USA). (a) The device provides four‐way steering and measures 65 cm, with a dual irrigation and a working channel of 1.2 mm. (b) The cholangioscope is passed through an 11F percutaneous sheath (red arrows) with a silicone‐coated valve fixed to the skin. The percutaneous sheath has an irrigation channel (blue arrows).

The 10.8F single‐use Spyglass Discover was used under endoscopy and fluoroscopy control. This short device allows diagnostic and therapeutic interventions in the biliary tree through a 1.2 mm working channel with a dual dedicated irrigation. The maneuverability and large angulation range allow targeted biopsies and accessing to difficult intrahepatic ducts for further therapy (Fig. 1). Notably, cholangioscopy‐guided electrohydraulic lithotripsy (EHL) using a dedicated generator (Autolith Touch, Boston Scientific; Walz Lithotron, Walz Elektronik, Rohrdorf, Germany) with 1.9–3F bipolar probes can be used in biliary stones, starting at low power, and increasing through medium and high at the discretion of the endoscopist. The cholangioscope can be used either through a percutaneous sheath with a silicone‐coated valve to facilitate the passage and theoretically decrease the risk of AEs, or directly though the percutaneous fistula.

The details of the technique, such as the wire‐guided or freehand cholangioscopy approaches, the presence of a water‐pump, the use of different device accessories (e.g. dedicated snare, basket, or forceps), and number of sessions were collected. The number of EHL probes were considered. The number of cholangioscopy examinations was noted. The procedure time, defined as the time from insertion to removal of the cholangioscope, was also collected.

### Outcomes

The primary outcome of this study was the clinical success, defined as the complete duct clearance in patients presenting with biliary stones, or the collection of histopathological samples by cholangioscopy‐guided biopsies (Spybite Max biopsy forceps; Boston Scientific) in the desired area for those patients presenting with indeterminate strictures or suspected malignant lesions.^21^ The accuracy of this last technique was assessed using as a gold standard the final histopathological diagnosis by other modalities (e.g. ERCP, surgery, etc.) or the retained benign/malignant status at the end of the follow‐up. Complete ductal clearance was retained when direct endoscopic cholangioscopy and cholangiography under fluoroscopy revealed no remaining stones. For indications other than biliary stones or indeterminate strictures, clinical success was not evaluated.

The secondary aims were the technical success and the AE rate. The technical success was retained when the Spyglass Discover was adequately advanced into the bile duct for proper intervention, as previously described.^11^ The AEs were evaluated following the standardized AGREE classification.^22^ The PTBD‐related and cholangioscopy‐related AEs were assessed separately. Delayed bleeding, acute cholangitis, and delayed perforation were considered. The length of hospitalization and clinical follow‐up were considered. Finally, procedure‐related mortality was noted.

### Statistical analysis

Categorical variables were expressed in percentages and compared using the *χ* ^2^‐test or Fisher's exact test. Normally and nonnormally distributed continuous variables are presented as mean ± SD or median (range), respectively. A two‐sided *t*‐test was used for continuous variables and a *P*‐value < 0.05 was considered statistically significant. SPSS software version 24 was used (IBM, SPSS, Chicago, IL, USA).

## RESULTS

### Patients

Fifty‐one patients (60.2 ± 15.9 years, 45.1% male) were included (Table 1). Patients with biliary stones had a higher prevalence of acute cholangitis compared to those presenting with indeterminate biliary strictures (81% vs. 52.9%), but this difference was not statistically significant (*P* = 0.065). Percutaneous cholangioscopy was performed in 11 patients with normal anatomy (21.6%) due to intrahepatic stones (*n* = 5) or intrahepatic indeterminate strictures (*n* = 4) not accessible to ERCP (two of them with Caroli disease), esophageal stenosis (*n* = 1), and a patient with a thoracic stomach (*n* = 1). Thus, most of patients with normal anatomy underwent percutaneous cholangioscopy due to intrahepatic diseases (*n* = 9, 81.8%). Overall, there were seven patients with previous failed ERCP (63.6%), two with a contraindication to ERCP (18.2%), and two who underwent a primary percutaneous approach (18.2%). Of 40 patients with altered anatomy (78.4%), there were only five with intrahepatic stones or strictures (12.5%).

**Table 1:** Baseline characteristics of 51 patients who underwent percutaneous cholangioscopy using a new short dedicated cholangioscope

Feature	Number (*n*, %)
Anatomy	
Normal	11 (21.6)
Roux‐en‐Y esophagojejunostomy	16 (31.4)
Roux‐en‐Y hepaticojejunostomy	14 (27.5)
Roux‐en‐Y choledochojejunostomy	5 (9.8)
Surgical gastrojejunostomy	3 (5.9)
Roux‐en‐Y gastric bypass	1 (2.0)
Scopinaro surgery	1 (2.0)
Indications	
Biliary stones	21 (41.2)
Indeterminate strictures	17 (33.3)
Biliary stones and stricture	13 (25.5)
Presence of acute cholangitis	35 (68.6)
Personal history of liver surgery	13 (25.5)

### Percutaneous cholangioscopy

Most of the PTBDs were performed using an internal–external drainage first, as shown in Table 2. The PTBD‐related complications were pneumothorax (*n* = 2), bile leak (*n* = 2), and biliary peritonitis (*n* = 2). All these complications were successfully managed with conservative treatment. The two patients who developed biliary peritonitis received antibiotics, requiring 3 and 4 days of extra hospitalization (AGREE grade II). Of note, one of them had this complication following percutaneous drain removal at the end of the whole procedure. Considering patients where a large percutaneous drain (≥10F) was used (*n* = 29, 56.9%), there was no statistically significant association with the rate of percutaneous‐related (3.4% vs. 22.7%, *P = *0.073) or cholangioscopy‐related complications (6.9% vs. 9.1%, *P* = 1). Patients with smaller percutaneous drains presented a higher overall complication rate (*n* = 5).

**Table 2:** Technical characteristics of percutaneous transhepatic biliary drainage (PTBD), performed prior to percutaneous endoscopic cholangioscopy

PTBD	Number (*n*, %)
Location	
Right intrahepatic duct	28 (54.9)
Left intrahepatic duct	22 (43.1)
Common bile duct	1 (2.0)
Type of percutaneous drain	
Internal–external	37 (72.5)
External	14 (27.5)
Size of first percutaneous drain	
7–8.5F	22 (43.1)
10–11F	21 (41.2)
12–15F	8 (15.7)
Exchange of percutaneous drain prior to cholangioscopy	20 (39.2)
Tract dilation prior to cholangioscopy	31 (60.8)
PTBD‐related complications	6 (11.8)

Percutaneous cholangioscopy was performed at a median of 8.5 days (range 0–373) from PTBD through a percutaneous sheath introducer of 11–14F (*n* = 36, 70.6%) or directly through the percutaneous fistula (*n* = 15, 29.4%) with or without preprocedural tract dilation. A water‐pump with saline was used (*n* = 49, 96.1%) and the median procedure time was 45 min (range 16–181). There were seven patients (13.7%) in whom PTDB and cholangioscopy were performed in the same session, most of them through a percutaneous sheath (*n* = 6). The radiologist assisted the procedure in 20 cases (39.2%) and a wire‐guided approach was used in 44 patients (86.3%). Of the remaining seven patients with a “freehand” introduction of the cholangioscope, cholangioscopy through a percutaneous catheter was performed in six cases (85.7%). Cholangioscopy‐guided EHL was performed in 29 cases (56.9%) using a median of one probe (range 1–2), in combination with a dedicated 15 mm retrieval basket (Boston Scientific) in eight cases (27.6%). Of note, in five patients with biliary stones, EHL was not necessary because the stone spontaneously migrated (*n* = 2) or was pushed with the cholangioscope into the jejunal lumen (*n* = 3). A median of one session (range 1–3) was needed for achieving a duct clearance (clinical success) in 28 cases (96.6%). There were only five patients requiring more than one session (17.2%).

**Figure 2:**
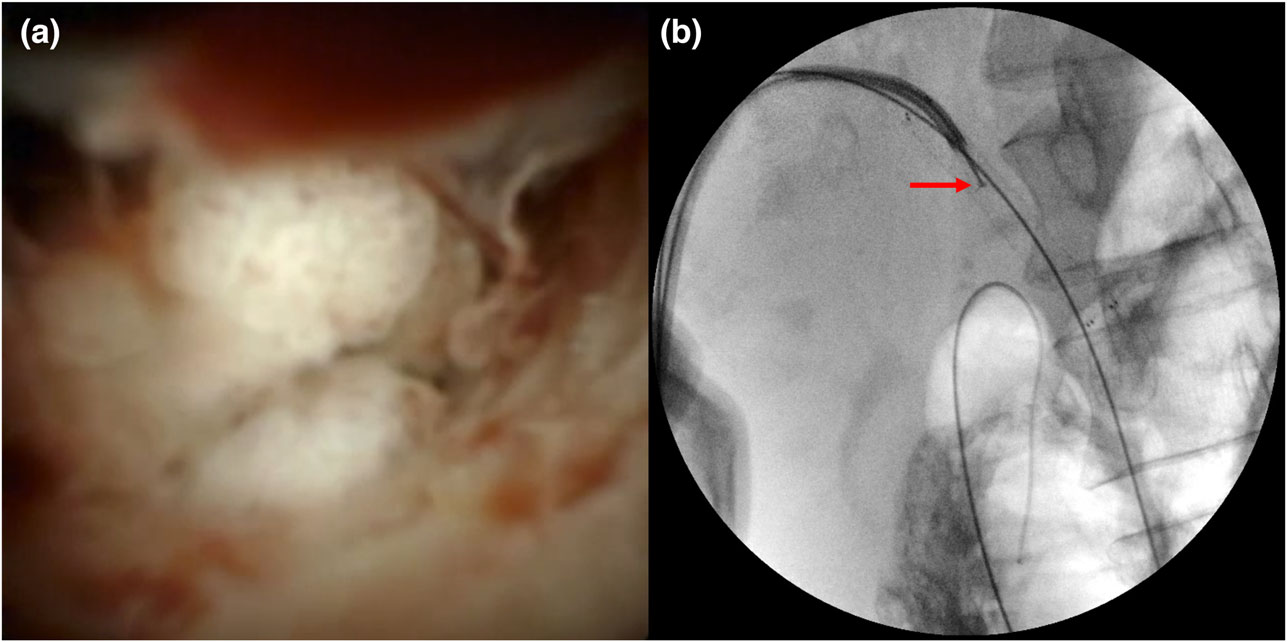
(a) Percutaneous cholangioscopy of a patient with an indeterminate biliary stricture. A nodular pattern with mucosal erythema and spontaneous bleeding was found. (b) Fluoroscopy image of a young male patient presenting with an indeterminate biliary stricture treated by previous percutaneous metal stenting. Cholangioscopy‐guided biopsies (red arrow) were performed.

Seventeen patients (33.3%) underwent cholangioscopy‐guided biopsies (Fig. 2), with targeted biopsies of the desired area in all cases (clinical success of 100%). There was only one nonconcordant patient with an indeterminate stricture and negative biopsies in whom an adenocarcinoma was diagnosed. Thus, the sensitivity, specificity, and accuracy of the technique were 85.7%, 100%, and 94.1%, respectively, in a median follow‐up of 9 months (range 1–23) for this subgroup of patients.

Overall, there were four cholangioscopy‐related complications (7.8%), all classified AGREE‐II. Three patients developed cholangitis and one patient who underwent percutaneous cholangioscopy directly through the percutaneous fistula with previous tract dilation developed biliary peritonitis. The median length of hospitalization was 8 days (range 1–41). The median follow‐up was 7 months (range 1–24). There was no procedure‐related mortality.

## DISCUSSION

In this multicentric retrospective study the efficacy and complications of a new short device for transhepatic cholangioscopy were evaluated. Out of 51 patients who underwent percutaneous endoscopic cholangioscopy using Spyglass Discover, the technical success was 100% and most of them presented with an altered anatomy (78.4%). The AE rate was 7.8% in a median follow‐up of 7 months. The clinical success for EHL was 96.6% requiring a median of one session, and the accuracy of cholangioscopy‐guided biopsies was 94.1%.

To the best of our knowledge, the present multicenter series represents the first study analyzing the feasibility and safety of this new hybrid technology. This technique is of major interest in patients with altered anatomy and difficult access by other routes such as EUS‐guided therapies. However, in our study 21.6% of patients had a normal anatomy, confirming that percutaneous cholangioscopy can also be useful in this setting for selected patients with intrahepatic diseases not accessible by conventional ERCP and even retrograde single‐operator cholangioscopy. Indeed, most of the patients with normal anatomy underwent percutaneous cholangioscopy due to intrahepatic diseases (81.8%), while only 12.5% of patients with altered anatomy had intrahepatic strictures or stones.

The clinical success of cholangioscopy‐guided EHL was high in patients with biliary stones but also intrahepatic stones with a more challenging access. Cholangioscopy‐guided biopsies also achieved a high accuracy of 94.1%. Both outcomes were higher compared to other series of peroral cholangioscopy.^21^ Easier manipulation of device accessories through a shorter cholangioscope with a wide range of angulation, as well as increasing experience with digital cholangioscopy itself, could have positively impacted these outcomes. However, it should be stressed that intrahepatic navigation in sectors other than those of PTBD has given access can also be challenging.

Although the percutaneous cholangioscopy AE rate was acceptable (7.8%), it is important to highlight that the overall AE rate per patient can be higher, particularly in patients needing several cholangioscopy sessions and/or percutaneous drain exchanges. Indeed, PTBD‐related complications occurred in 11.8% of patients in our study, thus the overall AE rate was 19.6%. However, all cholangioscopy‐related AE were classified as mild‐to‐moderate (AGREE‐II) and there was no mortality.

Compared to prior published studies using the Spyglass DS or DS2 systems^11^, ^13^, ^16^ that were designed for endoscopic bile duct access via a duodenoscope, our study included more patients and showed a comparable efficacy and AEs but fewer interventions per patient. The study by Gerges *et al*.^13^ included 28 patients, mainly with altered anatomy, and found a technical efficacy of 96% for the interventions and a histological accuracy of 100%. AEs occurred in 10.7%. In another study inserting a cholangioscope through a 12F sheath, in all five patients a technical success was achieved.^16^ In this regard, our data on a dedicated short cholangioscope for a transhepatic access highlight the efficacy and reduced rate of AEs. This may rely on the easier handling and higher degree of bending of this device. The 1.2 mm working channel with a dual dedicated irrigation and larger angulation range compared to other nondedicated ultraslim endoscopes previously used for percutaneous cholangioscopy could also have positively impacted our results.

In addition to the retrospective design, limitations of the present study were the different approaches for performing the technique (wire‐guided, through the percutaneous fistula, or through the sheath, etc.) in patients with different biliopancreatic anatomies and the different local protocols regarding the EHL settings and/or devices, as well as the number of cholangioscopy‐guided biopsies per procedure. However, our data more adequately reflect daily practice in this regard than monocentric studies from selected high‐volume centers. In addition, we did not evaluate the diagnostic accuracy in indeterminate strictures according to previous endoscopic classifications^23^ due to the nature of this pilot study and the fact that most of the included patients presented with biliary stones. Thus, the number of patients with isolated indeterminate strictures was too low to perform reliable analyses. However, the accuracy of cholangioscopy‐guided biopsies has been assessed in a relatively long follow‐up.

In conclusion, percutaneous cholangioscopy using this new short and dedicated device seems an effective technique for patients with biliary stones and/or indeterminate structures. This modality appears to be safe, with an acceptable AE rate, and requires a low number of sessions for achieving duct clearance or accurate diagnosis. Evidence‐based data assessing the best indications based on patient's characteristics (e.g. type of altered anatomy) and a new therapeutic algorithm with a focus on the standardization of the technique are lacking and should be the focus of further prospective studies.

## CONFLICT OF INTEREST

Author E.P.C.R. is a consultant for Boston Scientific and is an Associate Editor of *Digestive Endoscopy*. M.H. has received honoraria from Fujifilm. M.B. has received grants from Ovesco/Fides Medical and is a consultant for Taewoong – Prion Medical. S.V.M. is a consultant for Boston Scientific, Cook, Pentax, and Olympus. W.L. is a consultant for Boston Scientific and Cook. M.E. has received honoraria from Boston Scientific and is a consultant for Boston Scientific. G.W. is a consultant for Boston Scientific, Cook, and Pentax. The other authors declare no conflict of interest for this article.

## FUNDING INFORMATION

None.
